# Fast Photochemistry of Prototypical Phytochromes—A Species vs. Subunit Specific Comparison

**DOI:** 10.3389/fmolb.2015.00075

**Published:** 2015-12-23

**Authors:** Janne A. Ihalainen, Heikki Takala, Heli Lehtivuori

**Affiliations:** ^1^Department of Biological and Environmental Sciences, Nanoscience Center, University of JyväskyläJyväskylä, Finland; ^2^Department of Anatomy, Institute of Biomedicine, University of HelsinkiHelsinki, Finland; ^3^Department of Physics, Nanoscience Center, University of JyväskyläJyväskylä, Finland

**Keywords:** red photosensors, excited state dynamics, fluorescence, transient absorption, laser spectroscopy

## Abstract

Phytochromes are multi-domain red light photosensor proteins, which convert red light photons to biological activity utilizing the multitude of structural and chemical reactions. The steady increase in structural information obtained from various bacteriophytochromes has increased understanding about the functional mechanism of the photochemical processes of the phytochromes. Furthermore, a number of spectroscopic studies have revealed kinetic information about the light-induced reactions. The spectroscopic changes are, however, challenging to connect with the structural changes of the chromophore and the protein environment, as the excited state properties of the chromophores are very sensitive to the small structural and chemical changes of their environment. In this article, we concentrate on the results of ultra-fast spectroscopic experiments which reveal information about the important initial steps of the photoreactions of the phytochromes. We survey the excited state properties obtained during the last few decades. The differences in kinetics between different research laboratories are traditionally related to the differences of the studied species. However, we notice that the variation in the excited state properties depends on the subunit composition of the protein as well. This observation illustrates a feedback mechanism from the other domains to the chromophore. We propose that two feedback routes exist in phytochromes between the chromophore and the remotely located effector domain. The well-known connection between the subunits is the so-called tongue region, which changes its secondary structure while changing the light-activated state of the system. The other feedback route which we suggest is less obvious, it is made up of several water molecules ranging from the dimer interface to the vicinity of the chromophore, allowing even proton transfer reactions nearby the chromophore.

## Introduction

Phytochromes are red light-sensing photosensory proteins that exist in plants, fungi, and bacteria. The incident light leads to several structural and chemical changes of the protein, and thus, controls its biological activity. The structural changes between the two (thermodynamically stable) light-switchable states are considerably large in the photosensory module of the bacteriophytochromes (Takala et al., 2014a). The far-red fluorescence emission properties of phytochromes offer potential to tissue imaging (Fischer and Lagarias, 2004). Due to relatively low scattering, lower light absorption in living tissue, and good tissue penetration, the red light-sensing proteins provide an advantage over other photosensory proteins. The potential of phytochrome-based optogenetic switches have already been recognized by several laboratories (Shimizu-Sato et al., 2002; Möglich and Moffat, 2010; Piatkevich et al., 2013a,b; Gasser et al., 2014).

**Figure 1:**
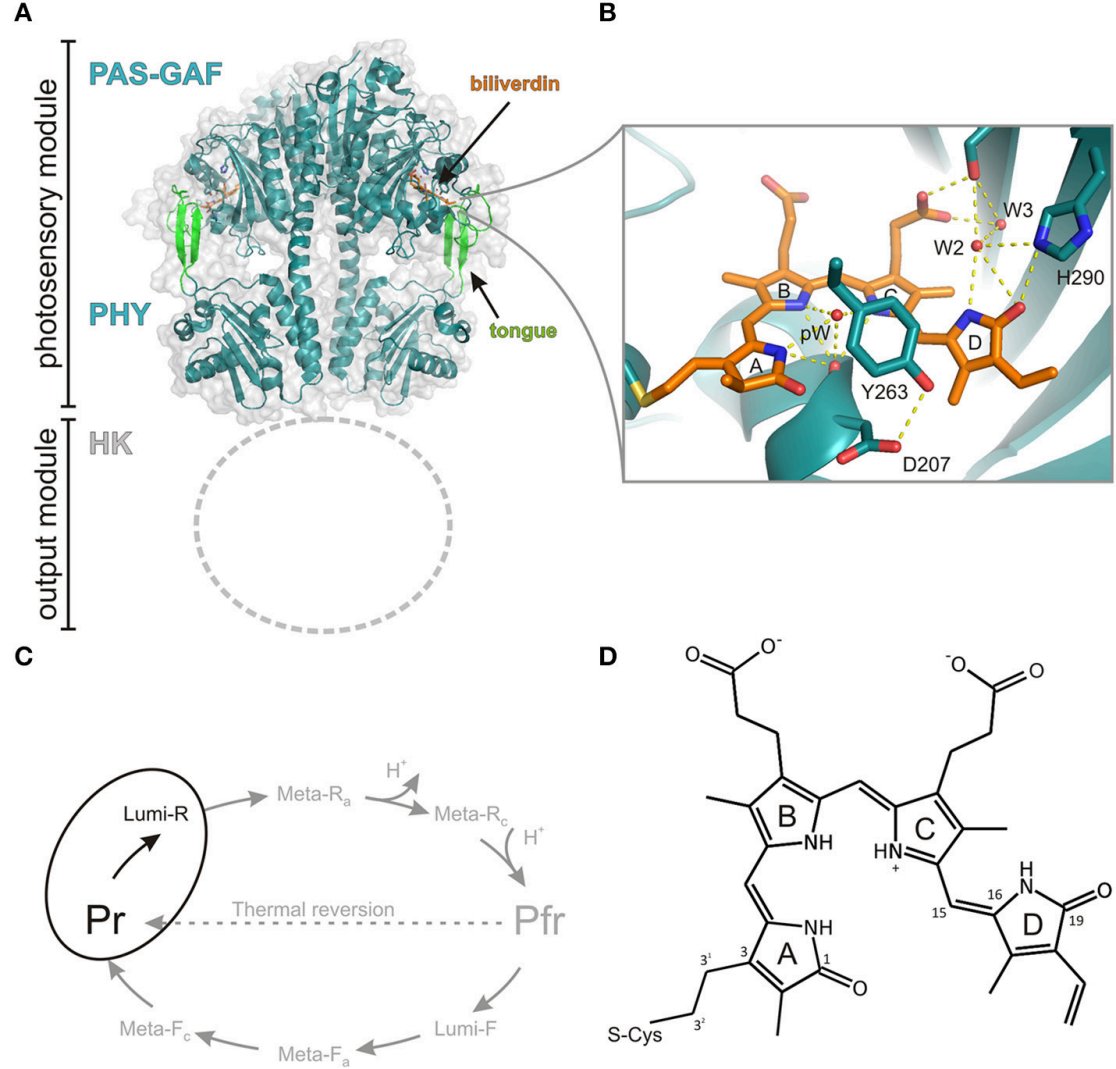
**Structure and photocycle of a canonical phytochrome from ***Deinococcus radiodurans***. (A)** The photosensory module of the phytochrome (PDB code 4O0P, Takala et al., 2014a) forms a parallel dimer that consists of chromophore-binding PAS and GAF domains, which are followed by a PHY domain. Due to the lack of structural information, the N-terminal histidine kinase (HK) domain is not shown. **(B)** A closed view of the biliverdin chromophore and its selected interactions to three water molecules pW (pyrrole water), W2, W3 and amino acids (Asp207, Tyr263, and His290). The panel is based on the high-resolution structure of the CBD fragment (PDB code 4Q0H, Burgie et al., 2014b). **(C)** Photocycle of the phytochrome with its intermediates. In this study, we concentrate on the first step of the forward reaction (Pr -> Lumi-R), highlighted in black. **(D)** The structure of the biliverdin molecule. The key atoms are numbered.

Phytochromes are widely found in the bacterial kingdom. A comprehensive description of various species, their occurrence, and function, is represented elsewhere (for example the review of Auldridge and Forest, 2011). On the other hand, the time-resolved spectroscopic studies of phytochromes have concentrated on a rather small set of phytochromes. We focus on phytochrome species which contain canonical domain architecture (Figure 1). We also concentrate on phytochromes whose light-activated reactions from Pr to Lumi-R have been studied on the ultra-fast time scales. These are the phytochromes from *Agrobacterium tumenfaciens* (*A. tumenfaciens*, Agp1), *Synechocystis sp*. PCC 6803 (cyanobacterial phytochrome, Cph1), *Deinococcus radiodurans* (*D. radiodurans, Dr*BphP), *Rhodopseudomonas palustris* (*R. palustris, Rp*BphP2 and *Rp*BphP3), and *Stigmatella aurantiaca, Sa*BphP1. The chromophore of the bacteriophytochrome is an open tetrapyrrole bilin molecule (Figure 1). In the case of Cph1, the chromophore is phycocyanobilin (PCB), The PCB differs from the BV by the lack of double bond character in the A ring and an ethyl-group in the C18 position. The plant phytochromes carry either PCB or phytochromobilin (PΦB) (Rockwell et al., 2006).

## Protein constituents and the photoactive states

A canonical bacteriophytochrome functions as a homodimer and consists of four different protein domains (Figure 1). The photosensory unit is made up of so-called PAS (PER, ARNT, SIM), GAF (cGMP phosphodiesterase, adenylate cyclase, FhlA), and PHY (Phytochrome-specific GAF related) domains. The PAS and GAF domains are together called a chromophore-binding domain (CBD). In prokaryotes, the bilin-binding residue resides in the PAS domain, whereas in cyanobacteria and plants the PCB and PΦB pigments are ligated with the GAF domain (Wagner et al., 2007). The fourth subunit, C-terminal of the PHY-domain, functions as biological effector and is often histidine kinase domain (HK). In addition to this canonical domain composition, a large set variation in the domain architecture exists in different phytochrome types. For example, *Synechocystis* Cph2 lacks the PAS domain, while cyanobacteriochromes lack both PAS and PHY domains and function as multi-subunit GAF domains (Rockwell et al., 2006).

The two photostable states of phytochromes are called Pr (red-absorbing state) and Pfr (far-red-absorbing state). The phytochromes with the Pr as dark resting state, like Agp1, Cph1, *Dr*BphP, *Rp*BphP2, and *Sa*BphP1, are called prototypical phytochromes. The bacteriophytochromes, like Agp2 from *A. tumenfaciens* and a phytochrome from *Pseudomonas aeruginosa* (*P. aeruginosa, Pa*BphP) thermally revert to the Pfr state and are called bathy phytochromes. In plants where a large variety of phytochrome isoforms exist, most of the phytochromes are prototypical.

The recent structural information has lifted the understanding about the phytochrome function considerably (Vierstra and Zhang, 2011; Burgie and Vierstra, 2014). The CBD fragment of *Dr*BphP was the first ever-published phytochrome structure in atomic resolution (Wagner et al., 2005, 2007). This structure confirmed the bilin-binding pocket and the conformation of BV as a ZZZ_ssa_ conformation (Figure 1) (Wagner et al., 2005). It revealed a peculiar figure-of-eight-knot structure which bridges the PAS and GAF domains. The refined CBD structure confirmed how C3^2^ in the vinyl group in the A-ring of the BV binds via a thioether linkage to the protein. Higher resolution structures revealed a number of coordinated water molecules and buried contacts between the monomeric units as dimerization sites (Wagner et al., 2007). Later, Auldridge et al. utilized this information for the production of monomeric CBD protein (Auldridge et al., 2012). Comparison with the CBD structures of other species set an important basis in the understanding about the photoconversion mechanism of the bilin molecules in the binding pocket (Yang et al., 2007). The high-resolution structures of CBD proteins have naturally been highly beneficial in the design of phytochrome-based near-infrared fluorescent proteins (Shu et al., 2009; Filonov et al., 2011; Auldridge et al., 2012; Shcherbakova and Verkhusha, 2013; Bhattacharya et al., 2014; Yu et al., 2014).

The structures of the full photosensory module (CBD-PHY) of Cph1 (Essen et al., 2008), *Pa*BphP (Yang et al., 2008, 2009), *Dr*BhP (Burgie et al., 2014a; Takala et al., 2014a), and a PhyB isoform from *Arabidopsis thaliana* (Burgie et al., 2014b) have been reported. The structure of the photosensory module resembles a tandem-GAF arrangement with a long connecting helix backbone (Essen et al., 2008). The PHY domain extends near to the chromophore by a so-called tongue-region which has a β-hairpin structure or an α-helical structure in the prototypical and bathy phytochromes, respectively, in their resting state (Essen et al., 2008; Yang et al., 2008). This tongue region contains a conserved PRxSF motif that interacts with the GAF domain near the chromophore and blocks the solvent accessibility to the chromophore-binding pocket. In the Pr state, this tongue motif forms a salt bridge between residues Asp207 and Arg466. The structural studies confirmed the15Za and the 15Ea conformations of the biliverdin in the Pr state and Pfr state, respectively. The BV isomerization leads to changes in the PHY-GAF interaction matrix. The β-hairpin structure in the tongue region disappears and an α-helical structure is stabilized. In the same process, a separation of the sister PHY domains was observed (Takala et al., 2014a). At the moment, the high-resolution structural information of the full-length phytochrome is missing and we need to settle for electron microscopic information (Burgie et al., 2014a,b). Solid-state magic-angle spinning NMR spectroscopy has also revealed detailed information about the hydrogen bond network around the chromophore (Song et al., 2011). Most of the studies have been conducted with Cph1 and oat PhyA proteins, however.

The kinetic information between the Pr and Pfr states relies on visible and vibrational spectroscopic results. The spectroscopic results are, however, difficult to link directly with the structural and chemical changes of the protein. The transition between Pr and Pfr state contains intermediate states (Figure 1), initially determined by UV-Vis absorption spectral changes at various temperatures (Eilfeld and Rüdiger, 1985). A similar method has also been used for the characterization of these states by the means of FTIR-spectroscopy (Foerstendorf et al., 2001; Schwinté et al., 2009; Piworski et al., 2010) and FT-Raman spectroscopy (Matysik et al., 1995). Due to the resonance-Raman conditions the signal assignment of the Raman spectra concentrates on the bilin vibrational modes. The FTIR-spectroscopy reveals information also from the protein and the assignment of IR-absorption spectrum is more challenging (Foerstendorf et al., 2001; Barth and Zscherp, 2002; Schwinté et al., 2009; Piworski et al., 2010; Stojkovicì et al., 2015; Velazquez Escobar et al., 2015). The clearest changes are in the 1730 cm^−1^ region, which reports the carbonyl vibrations of the chromophore. Several Amide I transitions have been indicated to the changes in the secondary structure of the protein during the reaction. The first intermediate, which is formed from the excited state bilin molecule is called Lumi-R state (Figure 1). It has a characteristic, slightly red-shifted absorption band. The transition between excited Pr^*^ to Lumi-R takes place in ps-ns range as it occurs via the excited state of the bilin molecule. The Pr to Lumi-R reaction is the gateway reaction to the photocycle (Figure 1). The quantum yield of the total Pr to Pfr photo reaction is mainly determined by the Pr to Lumi-R-reaction although a back reaction channel from Lumi-R to Pr state has been observed with a time-scale of 100 ns (Mathes et al., 2015). Typically, the fast photo processes are studied by means of ultrafast transient absorption techniques, either in the visible region or in the mid-infrared region, but also fluorescence techniques have been used for determining the excited state lifetimes. The description of this transition will come later in the ultra-fast spectroscopy section. The Lumi-R state transfers to so-called Meta-R_*a*_ state in about 100 μs time scale and shows a further red-shifted absorption. The next transition is Meta-R_a_ to Meta-R_c_ transition and it takes place in ms time scale, after which the protein undergoes the Meta-R_c_ to Pfr reaction. During these phases, kinetic proton transfer reactions take place (van Thor et al., 2001; Borucki et al., 2005). In the transition from Meta-R_a_ to Meta-R_c_ state a proton is released to the solvent which is again taken up by the protein in the Meta-Rc to Pfr reaction. The spectroscopic character during these reactions is a decrease of the extinction coefficient at most of the spectral region and a final far-red shift of the absorption. Thus, the decrease of the absorption intensity represents the proton release mechanism in the protein. The site(s) of the released and reclaiming site(s) of protons are unknown, however. A recent study suggests a model of the proton transfer pathway and a tautomeric system in bathy phytochromes (Velazquez Escobar et al., 2015), initially suggested by Lagarias and Rapoport (1980).

Probably due to crystal packing effects, the studies of intermediate states with crystallography-based techniques have been challenging. Up to present, the nature of the various intermediate states has been studied structurally by the means of cryotrapping X-ray crystallography (Yang et al., 2011). Detailed structural changes in the chromophore-binding pocket under illumination at the temperature range of −180°C to −120°C report the initial changes of the chromophore. Besides temperature-dependent experiments, rather extensive mutagenesis approaches have been linked to resonance Raman experiments. Several site-selective mutations in the vicinity of the chromophore (like in Asp207, Tyr263, His290, see Figure 1) or in the tongue region (e.g., Arg466) block the photocycle to a certain intermediate state, which can then be then probed by resonance Raman spectroscopy (Wagner et al., 2008).

## Ultra-fast kinetics of the Pr^*^ to Lumi-R -transition

**Figure 2:**
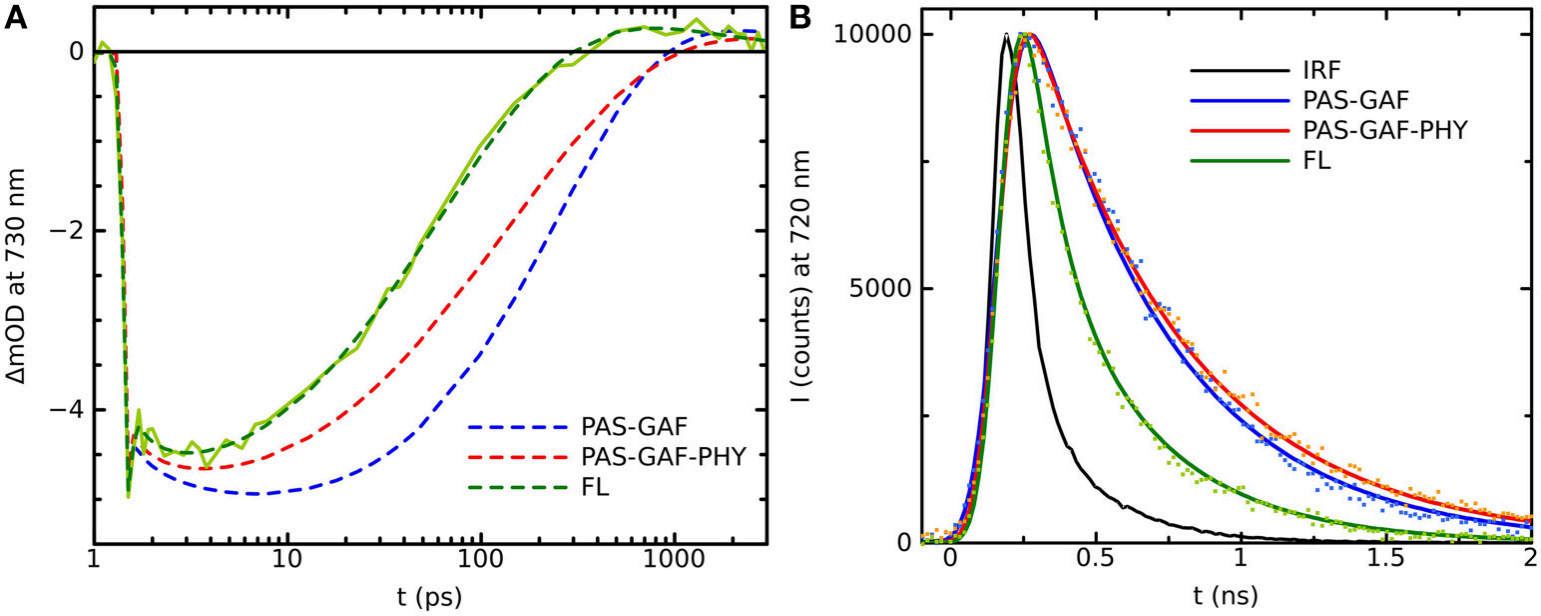
**(A)** Transition absorption decay traces of PAS-GAF (blue), PAS-GAF-PHY (red), and FL (green) from *Deinococcus radiodurans* excited at 656 nm and monitored at 730 nm. Solid lines show the multiexponential fit of the data, which result in following characteristic lifetimes of each complexes are: 1.5, ps and 320 ps for CBD; 1.2 ps and 170 ps for CBD-PHY; 0.5 ps and 70 ps for FL. **(B)** Emission decays of PAS-GAF, PAS-GAF-PHY, and FL from *Deinococcus radiodurans* excited at 660 nm and monitored at 720 nm. IRF is the instrument response function. The corresponding average lifetimes are: 410 ps for CBD; 550 ps for CBD-PHY; 340 ps for FL. The fluorescence quantum yields are 0.034, 0.017, and 0.008 for CBD, CBD-PHY, and FL, respectively. The samples were prepared as described in Takala et al. (2014b). The pump–probe technique for time-resolved absorption was used to detect fast processes with a time resolution shorter than 0.2 ps. A laser setup with an integrated one-box femtosecond Ti:sapphire laser (Quantronix Integra C) was used to pump two home-built non-collinear optical parametric amplifiers (NOPAs) to produce the (656 ± 14) nm pump pulses. A white light continuum generated in a sapphire crystal was used for probing. After the sample, the probe and reference beams entered a monochromator (Acton), and the detection was set to (730 ± 8) nm. Fluorescence decays of the samples in the nanosecond time scales were measured using a time-correlated single photon counting (TCSPC) system (PicoQuant GmBH). The excitation wavelengths were 660 nm. The monochromator (Jobin Yvon) was used to detect the emission at 720 nm with a single photon avalanche photodiode (MPD). To avoid excessive sample degradation in both time-resolved measurements, the sample solution was cycled using a peristaltic pump (Ismatec). A far-red diode at (750 ± 5) nm (Leading-Tech Laser Co.) was used to transform the sample to the Pr state by constantly illuminating. More details in Lehtivuori et al. (2013).

Plant phytochromes were the first phytochrome systems to be studied with ultra-fast spectroscopic methods (Sineshchekov, 1995). The initial photoprocesses of the oat phytochrome were determined to be around 30 ps. Similar photoactivated reaction times have been determined for cyanobacterial Cph1 (Heyne et al., 2002; van Thor et al., 2007; Kim et al., 2013). The time-resolved IR-spectroscopy (tr-IR) follows the most intimately of the structural changes of the chromophore and/or its protein environment in the Pr^*^ to Lumi-R reaction. Recently, by using polarized tr-IR experiments (Yang et al., 2012, 2014) elegantly recorded the orientation of C_19_ = *O* bond of the D-ring after photoexcitation and thus demonstrated the action of the Pr^*^ to Lumi-R reaction. The time constant for the rotation of the D-ring was reported being about 30 ps in Cph1Δ2 (CBD-PHY). In addition, two different PCB orientations were detected in the resting Pr state with significantly different H-bond networks and different rotation yields for both starting orientations (Yang et al., 2014). Longer reaction lifetimes have been reported for bacteriophytochromes, where the excited-state reactions were slower, about 100–300 ps (Toh et al., 2011a,b; Lehtivuori et al., 2013; Mathes et al., 2015). The Agp1 shows a 30 ps photoproduct formation (Schumann et al., 2007; Linke et al., 2013), which would indicate more similar lifetimes with the plant and cyanobacterial phytochromes. However, the lifetime results from *Dr*BphP, *Pa*BphP, and *Sa*BphP, are from truncated systems. By plotting the kinetics of the full-length system with the truncated constructs in (Figure 2), we show clearly longer decay times in the transient absorption data and fluorescence data of the shorter constructs than in the full-length system, in line with (Toh et al., 2011a,b; Lehtivuori et al., 2013; Mathes et al., 2015).

In ultra-fast spectroscopic studies, it has become clear that the excited state decay is complex with multi-exponential kinetics (Sineshchekov, 1995). The multi-exponential decay profiles indicate the multiple pathways of the excited Pr^*^ state, including sub-ps S1-relaxation processes, fluorescence, and (multiple) non-radiative (productive and non-productive) decay channels. By using two different excitation wavelengths and a rate distribution modeling, Heyne et al. observed a different type of excited-state kinetics for Cph1 (Heyne et al., 2002). Multi-pulse experiments in the transient absorption data have provided interesting details on the excited-state dynamics of the Pr^*^ states (Kim et al., 2013, 2014). In these experiments a “fluorescing” pool, non-radiative decay pool, and a reactive pathway with the time constants of the reactions between each of the pools are identified (Kim et al., 2014).

All of the above-mentioned studies indicate that the phytochrome systems contain strong non-productive channels. This has a consequence that the photochemical yield of the Pr^*^ to Lumi-R transition is low. In all studied species it has been shown to be between 0.1 and 0.2 for cyanobacterial phytochromes (Schumann et al., 2007; van Thor et al., 2007) and 0.05–0.15 for bacteriophytochromes (Toh et al., 2010; Mathes et al., 2015; Lehtivuori et al., unpublished).

In addition to the multiple decay pathways, the multi-exponential decay profile of the phytochromes may indicate the heterogeneity of the system. The heterogeneity vs. the homogeneity of the Pr state has been under debate the last decade. With NMR-studies (which probes solely the electronic ground states), Song et al. (2011) stated the presence of multiple Pr states in the Cph1 system, whereas the Pfr state is homogenous. Also low temperature single-molecule spectroscopic and site-specific fluorescence experiments indicate heterogeneity in the Pr state in several species (Nieder et al., 2009, 2013; Sineshchekov et al., 2014; Yang et al., 2014). In fact, Nieder et al. revealed, in addition to the heterogeneity between individual particles, spectral diffusion among single particles (Nieder et al., 2009). Thus, the phytochromes switch between the spectral forms even at very low temperatures. Room temperature Raman experiments with the Cph1 systems demonstrate the homogenous behavior of the absorption profile (Dasgupta et al., 2009; Spillane et al., 2009).

## Excited state lifetimes—time-resolved fluorescence studies

As mentioned above, a fraction of the excitation in the phytochrome system is emitted as fluorescence and phytochromes offer great potential for far-red fluorescent proteins (Fischer and Lagarias, 2004; Miller et al., 2006; Shu et al., 2009; Filonov et al., 2011; Auldridge et al., 2012; Shcherbakova and Verkhusha, 2013; Bhattacharya et al., 2014; Yu et al., 2014; Shcherbakova et al., 2015). However, regardless of the low photochemical yield, wild-type phytochromes are typically poorly fluorescent with fluorescence yields ranging from 0.01 to 0.04 (Fischer and Lagarias, 2004; Toh et al., 2010; Zienicke et al., 2011; Auldridge et al., 2012). Often, the low fluorescence yield is linked to photoisomerization activity (i.e., Pr^*^ to Lumi-R production). This, however, is a misconception as the largest decay channel for phytochromes systems are typically the non-productive channels (neither fluorescent nor Lumi-R-forming channel). This can be rationalized from the results of the fluorescence lifetime experiments. As stated above, the initial photoreaction of the cyanobacterial Cph1 take place in about 30 ps, but their fluorescence lifetimes have been measured to be around 1 ns (Otto et al., 2003; Miller et al., 2006). The same is true for plant systems (Sineshchekov, 1995). In bacteriophytochromes the difference between transient absorption and the transient fluorescence experiments is smaller (Figure 2) although in the case of Agp1 the photochemical reaction, from Pr^*^ to Lumi-R, is also around 30 ps (Schumann et al., 2007; Linke et al., 2013). The photoreaction times and fluorescence lifetimes of various species are gathered in the Table 1. At first glance, the difference between the reported photoreaction times and fluorescence lifetime, together with the low quantum yields, appear puzzling. In fact, fluoroproteins with excited-state lifetimes of about 2 ns and switching ability, would be good fluorescent proteins. For example, the excited state decay times of GFP are 2.8 and 3.3 ns (Striker et al., 1999) and the fluorescence yield can be as high as 0.8. In principle, the time-resolved fluorescence reflects the general lifetime of the excited state and reveals information about the initial photochemistry. There is, however, a caveat. By using ps laser pulses, which are typically used in single-photon counting set ups, only processes slower than about 100 ps are recorded. As fluorescence rates are considerably slower than the photochemical reactions of the phytochromes, only the fluorescence process is detected and other photo-activated processes remain underneath of the excitation pulse. In addition, other fluorescent channels, like the Lumi-R state (Sineshchekov, 1995), may influence to the lifetime experiments as the mixtures of Lumi-R and Pr fluorescent states are detected.

**Table 1:** **Excited state lifetimes of phytochrome systems from various species**.

**Species**	**Construct**	**Cof**	**Pr lifetime**	**Yield (%)**	**References**
Agp1	FL	BV	25 ps (ppf), 540 ps^*^ (flt)	9	Schumann et al., 2007; Linke et al., 2013
*Dr*BphP	PAS-GAF	BV	300 ps, 410 (flt)		Lehtivuori et al., 2013; Figure 2
*Dr*BphP	PAS-GAF-PHY	BV	170 ps, 550 (flt)		Figure 2
*Dr*BphP	FL	BV	70 ps, 340 (flt)		Figure 2
*Rp*BphP2	PAS-GAF	BV	175 ps		Toh et al., 2011a,b
*Rp*BphP2	PAS-GAF-PHY	BV	58 ps	13	Toh et al., 2011a,b
*Rp*BphP3	PAS-GAF	BV	300 ps		Toh et al., 2011a,b
*Rp*BphP3	PAS-GAF-PHY	BV	330 ps		Toh et al., 2011a,b
*Sa*BphP1	PAS-GAF	BV	225 ps		Mathes et al., 2015
*Sa*BphP1	PAS-GAF-PHY	BV	85 ps		Mathes et al., 2015
Cph1	PAS-GAF	PCB	30 ps (ppf)	15	Heyne et al., 2002
Cph1	PAS-GAF-PHY	PCB	25 ps (ppf)	13	Heyne et al., 2002; Yang et al., 2014
Cph1	FL	PCB	60 ps (ppf), 1.2 ns (flt)		Otto et al., 2003; Kim et al., 2014
Cph1	FL	PEB	ND(ppf) 3.2 ns (flt)		Otto et al., 2003
PhyB, Oat	FL	PCB	24 ps	15	Andel et al., 1997
PhyA, Oat	FL	PCB	24 ps		Müller et al., 2008
PhyA, Oat	65kDa	PCB	24 ps		Müller et al., 2008

*In many cases the decay is not single exponential. In cases where the average lifetime has not given, the lifetime has been estimated from the given analysis of the paper*.

* *Measured with locked BV, ppf, photoproduct formation; flt, fluorescence lifetime; Cof, Cofactor*.

## Discussion

We have summarized the key observations of the excited state reactions of the phytochrome systems. In the case of plant and cyanobacterial systems the excited-state reactions take place in about 30 ps and the fluorescence lifetime is above 1 ns. If the photoisomerization is impaired, the excited-state lifetimes can be increased up to 3.2 ns. In the bacteriophytochrome systems the photoreaction and excited-state decay processes have similar photoreaction excited state lifetimes, between 100 and 300 ps, and with site-selective mutations the lifetime can be increased to 870 ps (Bhattacharya et al., 2014). As the plant and cyanobacterial systems bind PCB and PΦB chromophores and the bacteriophytochromes BV chromophore, it is clear that the type of the pigment has a role in the excited state lifetime.

We emphasize, however, that the excited state reactions of the phytochromes are complex. Three processes, photo-isomerization, fluorescence, and non-photochemical quenching, are competitive, and we still lack a comprehensive picture of these reactions. One of the main stumbling blocks is the description of the interaction lattice of the bilin molecule with its environment. In the highest resolution structural models, obtained from the CBD systems (Wagner et al., 2007; Auldridge et al., 2012; Burgie et al., 2014b) the amino acid positions as well as the oxygen atoms of the water molecules are well-described. Different species show a different amino acid arrangement in the chromophore binding pocket (Mathes et al., 2015). It is, however, too straightforward to link an effect of single amino acid change in the structure directly to the excited state reaction, such as isomerization process. For example, the protonation states of the chromophore and its nearby histidine-residues influence the photochemical behavior of the molecule. Moreover, a labile protonation state can lead to several different conformations of the amino acids around the chromophore, and thus, heterogeneity in the system. Such effects are invisible in the X-ray crystal structures of the protein complexes.

**Figure 3:**
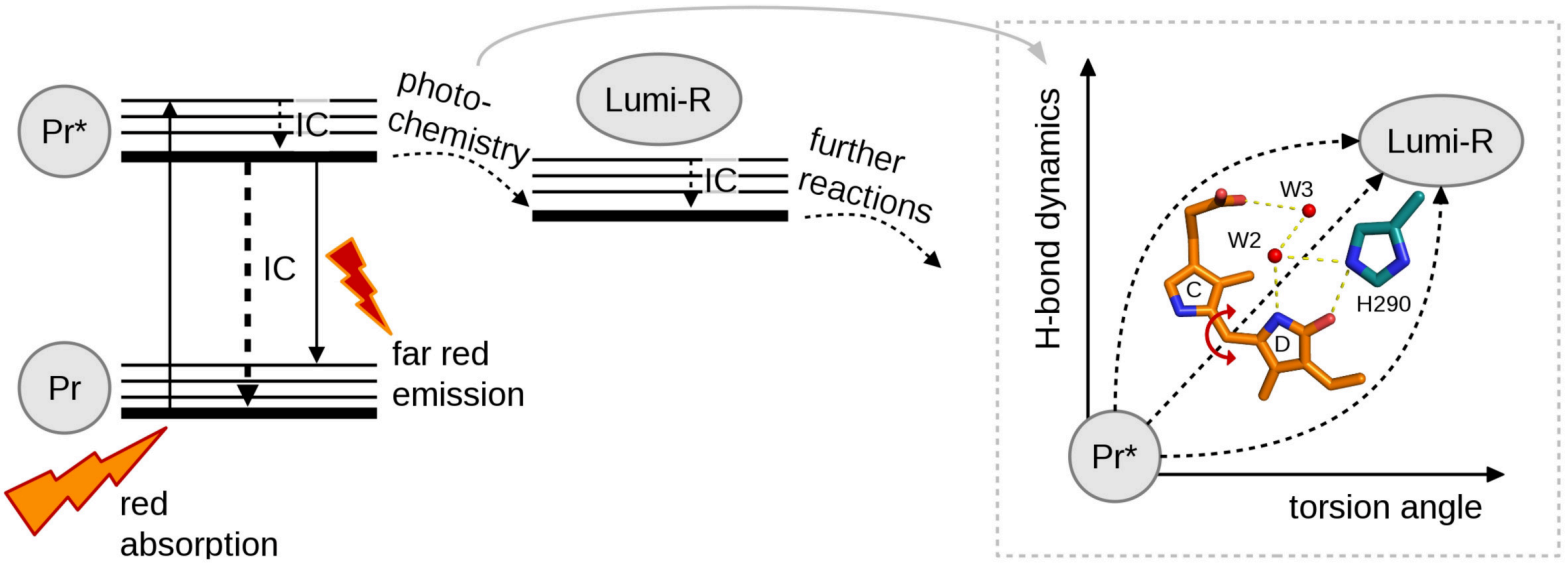
A simplified reaction diagram showing the excitation of molecule with red light from Pr ground state to its singlet excited state (Pr^*^) followed by internal conversion (IC), fluorescence or photochemistry. Pr^*^ relaxes back to the ground state by IC or far-red fluorescence. For photochemistry at least two coordinates are needed to describe the complete reaction, torsion angle and H-bond dynamics.

The great sensitivity of the excited state behavior of the chromophore makes possible for other subunits to influence the photochemical reactions of the chromophore from the larger distances. We have shown that on top of the variation in photo-excitation kinetics among different phytochrome species, each type of construct, i.e., the chromophore-binding domain (CBD), the photosensory core (CBD-PHY), or the full-length phytochrome, show differences in the excited state kinetics. We interpret this variation as the feedback mechanisms of the PHY and effector (HK) domains to the CBD domain. The first feedback route is the tongue of the PHY domain (Figure 1). In the Pr state, a salt bridge has formed between the Asp207 (from CBD) and Arg466 (in the PHY tongue) whereas in Pfr state the Asp207 coordinates with Ser468 of the tongue (Takala et al., 2014a). The Asp207, part of the conserved DIP motif, locates in the central position in the chromophore-binding pocket and locks the so-called pyrrole (pW in Figure 1B) water in its place. Furthermore, the interactions between the sister HK domains may stabilize the PHY domain orientation and further stabilize the chromophore binding pocket via tongue interactions. Moreover, the tongue controls solvent access to the chromophore-binding pocket. Thus, in the case of truncated CBD systems more water molecules occupy the chromophore binding pocket than in the CBD-PHY and FL-systems. The second feedback route could be a trail of water molecules from the protein interior to the nearby D-ring of the chromophore. In our opinion, the water lattices from the protein interior to the nearby chromophore have gained too little attention. The water molecule(s) nearby the NH-group and the CO group of the D-ring, marked as W2 and W3 in (Figure 1) and (Figure 3) certainly have H-bond character to the D-ring in the Pr-state and play role in the reaction to Lumi-R. These water molecules seem to be rather conserved in the structures of each species published to date and allow (water mediated) hydrogen-bonding network from deeper sites of the protein scaffold, and possibly, proton transfer pathways as well. Unfortunately, it is very demanding to perform a systematic study about the effect of the H-bond network of these water molecules. We propose that these differences are enough to build up slightly different micro-environment around the pigment in its ground and excited state so that it influences the excitation state kinetics of the systems.

## The reaction coordinates toward productive Lumi-R state

Full understanding about the photochemical reaction of phytochromes requires to reveal the most representative reaction coordinate along which the system proceeds from excited Pr^*^ state to Lumi-R state. Figure 3 summarizes the two main coordinates involved in reaction and how they are linked to structural changes. Temperature-dependent spectroscopic experiments have revealed a barrier along a reaction coordinate, with the activation energy of about 5 kJ/mol (Sineshchekov, 1995; Andel et al., 1996; Kim et al., 2013), which corresponds for example to the strength of one hydrogen bond in a system. By using temperature-dependent fluorescence measurements, a small barrier (2–3 kJ/mol) has been determined in a so-called Pre-Lumi-R to Lumi-R step in Cph1 (Sineshchekov et al., 2014). An obvious reaction coordinate would be the torsional rotation of the D-ring of the bilin chromophore. Rockwell et al. (2009) demonstrated by using circular dichroism spectroscopy that the C15 = C16 isomerization, or the rotation of the D-ring, occurs clockwise in the biliverdin phytochromes whereas the rotation is counter-clockwise in the phytobilin phytochromes (Rockwell et al., 2009). Just following the reaction coordinate of “torsion angle,” however, is not sufficient for describing the complete reaction. The other coordinate, called “H-bond dynamics” in (Figure 3), has actually many dimensions. The hydrogen bond network can be described between the D-ring and several amino acids and water molecules in its vicinity (Figure 3). Actually, rather similar amino acid composition around the D-ring (His290 in case of *D. radiodurans* and in Cph1, with an additional H-bond network of Lys183 and Ser297 in case of *Rp*BphP3, and diminished H-bonding character in the case of *Sa*BphP1) has been reported. Still, these species show different excited-state lifetimes (Table 1).

The non-productive channels of excitation energy are very dominant in all phytochrome systems and they are challenging to describe. Kennis and co-workers have put forward one potential pathway for excited state decay, namely an excited-state proton transfer reaction, which is suggested to take place among the pyrrole nitrogens of the chromophore, the pyrrole waters and their coordinating amino acid, Asp207 (Toh et al., 2010; Nieder et al., 2013). Other non-productive channels are most likely related to the tumbling of the D-ring, as its stabilization of the D-ring by the hydrogen bond network leads to stronger fluorescent molecules.

To increase the quantum yield of the fluorescence, internal conversion and photochemistry channels are to be diminished, either by protein mutations or by inserting chromophores with impaired photoisomerization capability (Shcherbakova et al., 2015). By using site-selective mutations for the stabilization of the chromophore D-ring environment has been shown to lead higher fluorescence quantum yields in bacteriophytochromes (Shu et al., 2009; Auldridge et al., 2012; Bhattacharya et al., 2014; Yu et al., 2014). An additional increase in the fluorescence yield may be obtained by rigidifying of the protein scaffold part (Bhattacharya et al., 2014). The photoisomerization pathway can be blocked by incorporating the apoprotein with phycoerythrobilin, PEB, which lack the double bond at the C15 = C16 position of the chromophore (Figure 1). In this case, the strain for isomerization is lost and excitation does not lead to the isomerization process. Another way of blocking the isomerization process is to use a BV15Za chromophore where C and D-rings are bridged with an additional linker preventing the rotation of the D-ring (Inomata et al., 2005). In these cases, stronger fluorescence with longer excited state lifetimes, up to 3.2 ns, have been reported for cyanobacterial and bacterial phytochromes (Heyne et al., 2002; Otto et al., 2003; Miller et al., 2006; Zienicke et al., 2011; Kim et al., 2014).

Finally, we would like to point out that the low quantum yields of the photoproductive states are critical only in the situations where low flux, or ultra-fast femtosecond pulses need to be used. For the studies of slower, thermally driven reactions, ns-laser pulses with sufficient excitation fluxes can be used. As the spectral shift of phytochrome is so large due to the light activated reaction, multiple excitation lead to full photo-conversion of the protein ensemble. With typical illumination systems with 5–20 nm spectral widths, photo-conversion yields of 0.6–0.7 are reached by constant illumination, which allows easily controlling the molecules for a large number of optogenetic purposes.

## Funding

Finnish Cultural foundation (for JI and HT, 0131067) and Academy of Finland (for HT and HL, 285461 and 277194, respectively) are acknowledged.

### Conflict of interest statement

The authors declare that the research was conducted in the absence of any commercial or financial relationships that could be construed as a potential conflict of interest.
